# Supplementary material for: Qualitative and Quantitative Real-Time PCR Methods for Assessing False-Positive Rates in Genetically Modified Organisms Based on the Microbial-Infection-Linked HPT Gene
Source: Int J Mol Sci. 2022 Sep 2;23(17):10000. doi: 10.3390/ijms231710000 (PMC9456445; doi:10.3390/ijms231710000)
Supplement: Supplementary file 1 [file ijms-23-10000-s001.zip › ijms-1853061-supplementary.pdf]

Table S1. 7259 bp isolated sequence from soybean sprouts

Labels: 5′ flanking sequence of *HPT* gene (5122 bp); *HPT* gene (1026 bp); 3′ flanking sequence of *HPT* gene (1111 bp).
ATCCGAGACCCTGCGGGTACTTACTCCTTTGACATACATTTCGGCGATGGCGAGCTTGA
GAGCACGCTCACTGCGCATGCCTTTCTCGATGCAGCTGGGATAAAACTCCATCCCGCG
AACTTGTGGTACTTTCAAGAGGACTTTCCCGGTCGCAAGATTGAATATTGGATTATTTG
GATTTACCAACAAACGCTACTATTTAGCTACGAAAACTCGAGAGAAGTACACAAAAC
CTTTTGGAAGTGATGGAAGTTTAAGTCCTCTTGATTATAGTGTAGGTATTGGAACAGGT
TTAGAATTAGGACATTTCCAGTTTATGTTTGGCTATGATTACCCTCTTAGTAACAGTTCG
AAAGATCCAGACCTAAGTCAAGTACGCCAACACAACTTCAGAGCAAGTATAGCATAT
TCATTTAGAAAGCTAAAAAAATAAAACTAACCTAGGGCTTTGGATATTTTCAAGGCAT
CTGATAAAGACTTAACATCATGGGTGCGGAGATAATCTGCACCTTTTTTGTATGCATAC
ATTTCTGCTGCAAGAGTTGGTGCTAAACGAGATTTCACATCGGTTCCAGTTATTTTACCT
AAGAATGATTTCCGTGACACTGCAATCATTACTTGCAAATTAAAAGCTTCTTGAATTTC
AGGGAAACGCTTCAAAACAAGAATAGATGTTTCTGGATTAGAGCCTAAAAAGAAGCC
CATACCCGGATCAAGAATAATTCGTTCACGCTTTACACCAGCCTCAACTAAAGCAGCA
ATTCTTTCTTTAAAAAATTCCATCATGGAAGTAAAAACCTCTTCCGGATTCGTTTCAAC
TTTAGTAGCTGCACCAATTCGCTGAACGGAGTGCATTAACACAAGTTTGCAATCTGAC
TTCGCCAAGCCTGAACAAATCTCAGGATAAGGAAAACCTTGAATATCATTAATAAAAT
CAACCTTTTGTTCTATGCAAAAACTCTGAACCTCAGGTTTAAATGTATCAACAGAAATA
GAAATGCCTTTTTCTTTTAAAGCCTTAATGACAGGTTTGAGTCTTTTGATTTCTTCCACA
ACGCCCACTTCAGTTGTATCAGGATTACTGGAAGCGGCTCCCAAATCAATCACATCTG
CTCCATCTTCAACCAAATGCAGAGCATGCTCAATTGCCTTATCTGTATCTAAATAAAGT
CCTCCATCGGAAAAACTATCGGTGGTTATATTTACGATTCCAAAAATCTTGCTCATATCA
TTTTGCTTTGATTACAAAAGTAGCATTTATCTTAATTGACTAATTATCACTTACGCAAATT
GACACATTTTCCCTATCTTTGCAGCCTAATTTTTTTGTTTTAATTCATTATGAAAACTGAT
AGGAACTGTCATTTCGAGTTTTTCGCGGTTTTGGGTCAAAATTCGGTTTTTTCCCCGAA
CTACGACTGTTTTCGCACCCCGAACAACTAATCCAATCAGAATTTTCCGGCAACGCCG
ACTCCAAGAAGTGTAATTCCAGTCTATTTCTGCCGTCAACCCCTGATTCGCGTTAATGC
GACAAAGCTCAAACACATAACTCCCCCACGAAGCCATATAAACTCCGATTTCGAGGT
GTTATCCTGTTCCGCATAAACTTCGTTTTTGGGTATTATACCTACACCAAAGCAAGAAG
ATTATGGGTCCAAACTTCATTTGTATATTTTGCAAACAAATATCTGCCTGTCATATCTGT
CGGAGTGTGGTGATACTCCTTTGCATTAAAAATGCGCTGATAAAAGGTAGTTCAGCAA
AAGGCGAAACGCCAATAAATATATCTATAACCGGTTTCGCCCGTTGATTGGGTATCGCC
GTGCTTCCGATATGTTCAATGGCTACGGCATGAGAACCGAAAAAATGGAGCAATTTTA
TTTTCTCTTCTTCAAAATTCTTTGTCCAATCCGGGTTATATTCCATAATTTCAATTTTCATG
ATAGTTTCGTCCTTGCCTCAAAAACCATGTTTGGTATTATACTCCATATCTCTCCGGTGC
AGGAATAGCCTTTTCTGATGCACGGCTAACAGCTTCCCATTCTTCCCAGGTAGCCATCC
GTGCTTTTAAGGTATCCATCGCCAAATCGTATACCATGCTGCCAAGGATAAGCCTCAAA
GGCGGATTATTGCTGGCTACTAATTTCATAAGGGCTTCCGCTGCCAAGGAAGGATCACT
ATCGACCGAATCTTCGGAATACTGTTTCGCCAGCTCATCGCGCAGTGTACCGTATGAAT
CTAATGGATTGCTGTAACTCATCGAAGTGTATAAATCTGTCCAATATCCTCCTGGTTCCA
CCATAGTGAGTTTCACTCCGAAATGTTCCGCTTCTTTTGCTAAGGCTTCACTCATTCCTT
CCAGCGCAAATTTACTTGCACTGTAAATACCAGACATCGGACCCGAGATAATAGCGCC

AATGCTTGTAATCTGTATGATATGTCCAGAACGCTGCGACCTCAAATAGGGCATCACTG
CCTGACAAACCCAAAGAGCTCCAAAAAAGTTTGTGTCCATTAGTTTCCGAGCATCGGA
TTCGTTTAGTTCTTCAATCATACCCATAGTCATGATACCCGCATTATTAACAACAATATC
AAGCCTACCGAAATGTTTAACTGCTGTTTCAACCGTAGAAAAAACAGCCTCCCTATCT
GTAACATCGAGGTTTAATGGCAGTAAGCTCTCTTGATATGTTTCTTCTAGCTTCGCCAAA
TTATCGATAGTCCTTGCAACTGCAACAACTTTATCCCCGGCTTTCAAGGCGGCGCATGT
AAAAGCATAGCCAAGCCCCTTGCTTGCTCCGGTAATAAACCATACTCTTTTATTTTCGC
TATTAGGTATCATAGATATTCTCCTTTAGGCTATTATAGAAAGATTATTATAAGCGAAATT
AATACCGTTAAACGCCATGAGCCCGACAATTTTGAGCACTTTGTTTTGTACTCCCAGAA
GCTCACCACTACAAATTATCATCAACCCGGTCCTTTTTTCAGTCCACTTGCGGTTTCATT
GTTACTTTGTCATAGCGTCGTGTCAGAATGCGGCAGAGAACAGCGTTTACTATCAACG
CAACACCAGACCAGAAAAACCATGTATTAACACCTATAACCTCAACAACCGGACCTG
CAACAAGTAAGCCTATCGGCATAGAAAGAGTCATGGCGGTCATCAAAAGGAAAAC
ACCTTGCCCATCATTTCAGGGGCAATGCTTTCTTGAACATAAGCCATAACAGGAACATT
CATAAATGGGCACTGTTGCAAAGTTAGCGATGAGGCAGCCTTTTGTCTTATTCAAAGG
CCTTACATTTCAAAAACTCTGCTTACCAGGCGCATTTCGCCCAGGGGATCACCATAATA
AAATGCTGAGGCCTGGCCTTTGCGTAGTGCACGCATCACCTCAATACCTTTGATGGTG
GCGTAAGCCGTCTTCATGGATTTAAATCCCAGCGTGGCGCCGATTATCCGTTTCAGTTT
GCCATGATCGCATTCAATCACGTTGTTCCGGTACTTAATCTGTCGGTGTTCAACGTCAG
ACGGGCACCGGCCTTCGCGTTTGAGCAGAGCAAGCGCGCGACCATAGGCGGGCGCTT
TATCCGTGTTGATGAATCGTGGGATCTGCCACTTCTTCACGTTGTTGAGGATTTTACCCA
GAAACCGGTATGCAGCTTTGCTGTTACGACGGGAGGAGAGATAAAAATCGACAGTGC
GGCCCCGGCTGTCGACGGCCCGGTACAGATACGCCCAGCGGCCATTGACCTTCACGTA
GGTTTCATCCATGTGCCACGGGCAAAGATCGGAAGGGTTACGCCAGTACCAGCGCAG
CCGTTTTTCCATTTCAGGCGCATAACGCTGAACCCAGCGGTAAATCGTGGAGTGATCG
ACATTCACTCCGCGTTCAGCCAGCATCTCCTGCAGCTCACGGTAACTGATGCCGTATTT
GCAGTACCAGCGTACGGCCCACAGAATGATGTCACGCTGAAAATGCCGGCCTTTGAA
TGGGTTCATGTGCAGCTCCATCAGCAAAAGGGGATGATAAGTTTATCACCACCGACTA
TTTGCAACAGTGCCGTTGATCGTGCTATGATCGACTGATGTCATCAGCGGTGGAGTGC
AATGTCGTGCAATACGAATGGCGAAAAGCCGAGCTCATCGGTCAGCTTCTCAACCTTG
GGGTTACCCCCGGCGGTGTGCTGCTGGTCCACAGCTCCTTCCGTAGCGTCCGGCCCCT
CGAAGATGGGCCACTTGGACTGATCGAGGCCCTGCGTGCTGCGCTGGGTCCGGGAGG
GACGCTCGTCATGCCCTCGTGGTCAGGTCTGGACGACGAGCCGTTCGATCCTGCCACG
TCGCCCGTTACACCGGACCTTGGAGTTGTCTCTGACACATTCTGGCGCCTGCCAAATGT
AAAGCGCAGCGCCCATCCATTTGCCTTTGCGGCAGCGGGGCCACAGGCAGAGCAGAT
CATCTCTGATCCATTGCCCCTGCCACCTCACTCGCCTGCAAGCCCGGTCGCCCGTGTCC
ATGAACTCGATGGGCAGGTACTTCTCCTCGGCGTGGGACACGATGCCAACACGACGC
TGCATCTTGCCGAGTTGATGGCAAAGGTTCCCTATGGGGTGCCGAGACACTGCACCAT
TCTTCAGGATGGCAAGTTGGTACGCGTCGATTATCTCGAGAATGACCACTGCTGTGAG
CGCTTTGCCTTGGCGGACAGGTGGCTCAAGGAGAAGAGCCTTCAGAAGGAAGGTCCA
GTCGGTCATGCCTTTGCTCGGTTGATCCGCTCCCGCGACATTGTGGCGACAGCCCTGG
GTCAACTGGGCCGAGATCCGTTGATCTTCCTGCATCCGCCAGAGGCGGGATGCGAAG
AATGCGATGCCGCTCGCCAGTCGATTGGCTGAGCTCATGAGCGGAGAACGAGATGAC
GTTGGAGGGGCAAGGTCGCGCTGATTGCTGGGGCAACACGTGGAGCGGATCGGGGAT

TGTCTTTCTTCAGCTCGCTGATGATATGCTGACGCTCAATGCCGTTTGGCCTCCGACTA
ACGAAAATCCCGCATTTGGACGGCTGATCCGATTGGCACGGCGGACGGCGAATGGCG
GAGCAGACGCTCGTCCGGGGGCAATGAGATATGAAAAAGCCTGAACTCACCGCGAC
GTCTGTCGAGAAGTTTCTGATCGAAAAGTTCGACAGCGTCTCCGACCTGATGCAGCTC
TCGGAGGGCGAAGAATCTCGTGCTTTCAGCTTCGATGTAGGAGGGCGTGGATATGTCC
TGCGGGTAAATAGCTGCGCCGATGGTTTCTACAAAGATCGTTATGTTTATCGGCACTTT
GCATCGGCCGCGCTCCCGATTCCGGAAGTGCTTGACATTGGGGAGTTTAGCGAGAGCC
TGACCTATTGCATCTCCCGCCGTGCACAGGGTGTCACGTTGCAAGACCTGCCTGAAAC
CGAACTGCCCGCTGTTCTACAACCGGTCGCGGAGGCTATGGATGCGATCGCTGCGGCC
GATCTTAGCCAGACGAGCGGGTTCGGCCCATTCGGACCGCAAGGAATCGGTCAATAC
ACTACATGGCGTGATTTCATATGCGCGATTGCTGATCCCCATGTGTATCACTGGCAAAC
TGTGATGGACGACACCGTCAGTGCGTCCGTCGCGCAGGCTCTCGATGAGCTGATGCTT
TGGGCCGAGGACTGCCCCGAAGTCCGGCACCTCGTGCACGCGGATTTCGGCTCCAAC
AATGTCCTGACGGACAATGGCCGCATAACAGCGGTCATTGACTGGAGCGAGGCGATG
TTCGGGGATTCCCAATACGAGGTCGCCAACATCTTCTTCTGGAGGCCGTGGTTGGCTTG
TATGGAGCAGCAGACGCGCTACTTCGAGCGGAGGCATCCGGAGCTTGCAGGATCGCC
ACGACTCCGGGCGTATATGCTCCGCATTGGTCTTGACCAACTCTATCAGAGCTTGGTTG
ACGGCAATTTCGATGATGCAGCTTGGGCGCAGGGTCGATGCGACGCAATCGTCCGATC
CGGAGCCGGGACTGTCGGGCGTACACAAATCGCCCGCAGAAGCGCGGCCGTCTGGA
CCGATGGCTGTGTAGAAGTACTCGCCGATAGTGGAAACCGACGCCCCAGCACTCGTC
CGAGGGCAAAGGAATAGAGTAGATGCCGACCGAACAAGAGCTGATTTCGAGAACGC
CTCAGCCAGCAACTCGCGCGAGCCTAGCAAGGCAAATGCGAGAGAACGGCCTTACG
CTTGGTGGCACAGTTCTCGTCCACAGTTCGCTAAGCTCGCTCGGCTGGGTCGCGGGAG
GGCCGGTCGCAGTGATTCAGGCCCTTCTGGATTGTGTTGGTCCCCAGGGCACGATTGT
CATGCCCACGCACTCGGGTGATCTGACTGATCCCGCAGATTGGAGATCGCCGCCCGTG
CCTGCCGATTGGGTGCAGATCTTGCGCAACGAGATGCCGGCATACGATCCGCAAACC
ACACCAACTCGGAACATGGGTGCAGTGGCTGAATTGTTCCGGGCGTGTCGCAAACAC
TTTTGGAGGCAGCTATCACCCAGCCAAGGCCGGTATTTTCATGCTTTGTTAACCTTTTG
CAGTCCGAAGAGGCGCGCAAGCAAATCATTCTGGTCATTGACGGTCCAGCCGCCTGA
AAAGCCAAAGCCCTTCCGCAGCCACAGCATCGCCTCGAAACCCGCGATGGTTCGCCG
CGCCGTGTTAAAGGATTGGAAACCGCCGATCTTCGGCATGTTCTTCTTCACCCGGAAA
TGGTCGCTCTCAATCCCCTGCTGGAGATGTTTGGTCACATAATGCACGGGATCGGGATG
GAGAAGCCCATCATCAACCGACGTTTTGATCGTTGACGGGAAGGTGTTGGCCCCGTCC
GTCCCAATCCTGTTCGGCGACAACAAGGGTTCATCTTTAAGCATCTTTCGGAAGAACC
GCTTGGCAGCGTCGAGATCGCGCTTAGCGGTCAGCAGGAAATCCACCGGATTGCCAT
GCTTATCGATGGCTCGGTACAGATAGCGCCATTTGCCGCGGATCTTGACATAGGTCTCA
TCAATCCGGACCGAGCCGCAATGGGGTCGACGAAACTGCCGCAGCCGTTTCTCGATG
ACAGGTGCATAAGCCAATACCCAGCGGTTGATCGTGCTATGATCGACCTCGAAGCCCC
GCTCGCGAAACATCTCTTCCAAGTCACGATAGCTGAGCGGGTAGCGCAAGTACCAGG
CAACCGCCTGTACAATCAGCCAGACAGTCCAAGT

Table S2. 3207 bp sequence integrated into the plasmid vector pUC57.

Labels: 5′ flanking sequence of *HPT* gene (2922 bp); incomplete *HPT* gene (285 bp).
CTGTTTCGCCAGCTCATCGCGCAGTGTACCGTATGAATCTAATGGATTGCTGTAACTCA
TCGAAGTGTATAAATCTGTCCAATATCCTCCTGGTTCCACCATAGTGAGTTTCACTCCG
AAATGTTCCGCTTCTTTTGCTAAGGCTTCACTCATTCCTTCCAGCGCAAATTTACTTGCA
CTGTAAATACCAGACATCGGACCCGAGATAATAGCGCCAATGCTTGTAATCTGTATGAT
ATGTCCAGAACGCTGCGACCTCAAATAGGGCATCACTGCCTGACAAACCCAAAGAGC
TCCAAAAAAGTTTGTGTCCATTAGTTTCCGAGCATCGGATTCGTTTAGTTCTTCAATCAT
ACCCATAGTCATGATACCCGCATTATTAACAACAATATCAAGCCTACCGAAATGTTTAA
CTGCTGTTTCAACCGTAGAAAAAACAGCCTCCCTATCTGTAACATCGAGGTTTAATGG
CAGTAAGCTCTCTTGATATGTTTCTTCTAGCTTCGCCAAATTATCGATAGTCCTTGCAAC
TGCAACAACTTTATCCCCGGCTTTCAAGGCGGCGCATGTAAAAGCATAGCCAAGCCCC
TTGCTTGCTCCGGTAATAAACCATACTCTTTTATTTTCGCTATTAGGTATCATAGATATTC
TCCTTTAGGCTATTATAGAAAGATTATTATAAGCGAAATTAATACCGTTAAACGCCATG
AGCCCGACAATTTTGAGCACTTTGTTTTGTACTCCCAGAAGCTCACCACTACAAATTAT
CATCAACCCGGTCCTTTTTTCAGTCCACTTGCGGTTTCATTGTTACTTTGTCATAGCGTC
GTGTCAGAATGCGGCAGAGAACAGCGTTTACTATCAACGCAACACCAGACCAGAAA
AACCATGTATTAACACCTATAACCTCAACAACCGGACCTGCAACAAGTAAGCCTATCG
GCATAGAAAGAGTCATGGCGGTCATCAAAAGGGAAAACACCTTGCCCATCATTTCAG
GGGCAATGCTTTCTTGAACATAAGCCATAACAGGAACATTCATAAATGGGCACTGTTG
CAAAGTTAGCGATGAGGCAGCCTTTTGTCTTATTCAAAGGCCTTACATTTCAAAAACTC
TGCTTACCAGGCGCATTTCGCCCAGGGGATCACCATAATAAAATGCTGAGGCCTGGCC
TTTGCGTAGTGCACGCATCACCTCAATACCTTTGATGGTGGCGTAAGCCGTCTTCATGG
ATTTAAATCCCAGCGTGGCGCCGATTATCCGTTTCAGTTTGCCATGATCGCATTCAATC
ACGTTGTTCCGGTACTTAATCTGTCGGTGTTCAACGTCAGACGGGCACCGGCCTTCGC
GTTTGAGCAGAGCAAGCGCGCGACCATAGGCGGGCGCTTTATCCGTGTTGATGAATCG
TGGGATCTGCCACTTCTTCACGTTGTTGAGGATTTTACCCAGAAACCGGTATGCAGCTT
TGCTGTTACGACGGGAGGAGAGATAAAAATCGACAGTGCGGCCCCGGCTGTCGACGG
CCCGGTACAGATACGCCCAGCGGCCATTGACCTTCACGTAGGTTTCATCCATGTGCCA
CGGGCAAAGATCGGAAGGGTTACGCCAGTACCAGCGCAGCCGTTTTTCCATTTCAGGC
GCATAACGCTGAACCCAGCGGTAAATCGTGGAGTGATCGACATTCACTCCGCGTTCAG
CCAGCATCTCCTGCAGCTCACGGTAACTGATGCCGTATTTGCAGTACCAGCGTACGGC
CCACAGAATGATGTCACGCTGAAAATGCCGGCCTTTGAATGGGTTCATGTGCAGCTCC
ATCAGCAAAAGGGGATGATAAGTTTATCACCACCGACTATTTGCAACAGTGCCGTTGA
TCGTGCTATGATCGACTGATGTCATCAGCGGTGGAGTGCAATGTCGTGCAATACGAAT
GGCGAAAAGCCGAGCTCATCGGTCAGCTTCTCAACCTTGGGGTTACCCCCGGCGGTGT
GCTGCTGGTCCACAGCTCCTTCCGTAGCGTCCGGCCCCTCGAAGATGGGCCACTTGGA
CTGATCGAGGCCCTGCGTGCTGCGCTGGGTCCGGGAGGGACGCTCGTCATGCCCTCGT
GGTCAGGTCTGGACGACGAGCCGTTCGATCCTGCCACGTCGCCCGTTACACCGGACCT
TGGAGTTGTCTCTGACACATTCTGGCGCCTGCCAAATGTAAAGCGCAGCGCCCATCCA
TTTGCCTTTGCGGCAGCGGGGCCACAGGCAGAGCAGATCATCTCTGATCCATTGCCCC
TGCCACCTCACTCGCCTGCAAGCCCGGTCGCCCGTGTCCATGAACTCGATGGGCAGGT
ACTTCTCCTCGGCGTGGGACACGATGCCAACACGACGCTGCATCTTGCCGAGTTGATG
GCAAAGGTTCCCTATGGGGTGCCGAGACACTGCACCATTCTTCAGGATGGCAAGTTGG

TACGCGTCGATTATCTCGAGAATGACCACTGCTGTGAGCGCTTTGCCTTGGCGGACAG
GTGGCTCAAGGAGAAGAGCCTTCAGAAGGAAGGTCCAGTCGGTCATGCCTTTGCTCG
GTTGATCCGCTCCCGCGACATTGTGGCGACAGCCCTGGGTCAACTGGGCCGAGATCCG
TTGATCTTCCTGCATCCGCCAGAGGCGGGATGCGAAGAATGCGATGCCGCTCGCCAGT
CGATTGGCTGAGCTCATGAGCGGAGAACGAGATGACGTTGGAGGGGCAAGGTCGCGC
TGATTGCTGGGGCAACACGTGGAGCGGATCGGGGATTGTCTTTCTTCAGCTCGCTGAT
GATATGCTGACGCTCAATGCCGTTTGGCCTCCGACTAACGAAAATCCCGCATTTGGAC
GGCTGATCCGATTGGCACGGCGGACGGCGAATGGCGGAGCAGACGCTCGTCCGGGG
GCAATGAGATATGAAAAAGCCTGAACTCACCGCGACGTCTGTCGAGAAGTTTCTGATC
GAAAAGTTCGACAGCGTCTCCGACCTGATGCAGCTCTCGGAGGGCGAAGAATCTCGT
GCTTTCAGCTTCGATGTAGGAGGGCGTGGATATGTCCTGCGGGTAAATAGCTGCGCCG
ATGGTTTCTACAAAGATCGTTATGTTTATCGGCACTTTGCATCGGCCGCGCTCCCGATTC
CGGAAGTGCTTGACATTGGGGAGTTTAGCGAGAGCCTGACCTATTGCATCTCCCGCCG
TGCA

Table S3. Primers and probes used in this study.

Purpose	Name	Sequences (5'-3')	Amplicon size	Specificity
Screening of *HPT* gene	qHptF286	CAGGGTGTCACGTTGCAAGA	110 bp	*HPT* gene from GM crops
	qHptR395	CCGCTCGTCTGGCTAAGATC		
	QHptFP308 *	TGCCTGAAACCGAACTGCCCGCTG		
	HptF226	GAAGTGCTTGACATTGGGGAGT	472 bp	
	HptR697	AGATGTTGGCGACCTCGTATT		
GenomeWalker kit adaptor primers	AP1	GTAATACGACTCACTATAGGGC		GenomeWalker kit adaptor
	AP2	ACTATAGGGCACGCGTGGT		
Isolation of surrounding sequence of *HPT* gene	wHPT1-1	GCCGATGCAAAGTGCCGATAAACATAA		*HPT* gene
	wHPT1-2	TAGAAACCATCGGCGCAGCTATTTACC		
	wHPT1+1	CTATCAGAGCTTGGTTGACGGCAATT		
	wHPT1+2	TTGGTTGACGGCAATTTCGATGATGC		
	wHRSUW1-1	AATCAACCGAGCAAAGGCATGACCGAC		5' flanking sequence of *HPT*
	wHRSUW1-2	TGAAGGCTCTTCTCCTTGAGCCACCTG		
	wHRSUW2-1	GTTTCTGGGTAAAATCCTCAACAACGT		
	wHRSUW2-2	TCAACAACGTGAAGAAGTGGCAGATCC		
	wHRSUW3-1	CTCTTTCTATGCCGATAGGCTTACTTG		
	wHRSUW3-2	CCGGTTGTTGAGGTTATAGGTGTTAAT		
	wHRSUW4-1	GATGGAGGACTTTATTTAGATACAGAT		
	wHRSUW4-2	ACTGAAGTGGGCGTTGTGGAAGAAATC		
	wHRSDW1+1	TCGATGACAGGTGCATAAGCCAATACC		3' flanking sequence of *HPT*
	wHRSDW1+2	TCCAAGTCACGATAGCTGAGCGGGTAG		
Qualitative PCR analysis	Ghpt-F3	CGCTGATGATATGCTGACGC	261 bp	junction site between 5'-plasmid DNA sequence and *HPT*
	Ghpt-R3	GCCCTCCTACATCGAAGCTG		
qPCR analysis	Ghpt-QF3	CTCCGACTAACGAAAATC	156 bp	
	Ghpt-QR3	GTCGAACTTTTCGATCAG		
	Ghpt-QP *	ACGGCTGATCCGATTGGCAC		

* The TaqMan probes were labelled with 5′ FAM and 3′ BHQ1.

select all *100 sequences selected* GenBank Graphics Distance tree of results New MSA Viewer

	Description	Scientific Name	Max Score	Total Score	Query Cover	E value	Per. Ident	Acc. Len	Accession
☑	Klebsiella pneumoniae MH15-269M plasmid pMH15-269M_1 DNA, complete sequence	Klebsiella pneu…	13313	16954	99%	0.0	99.82%	288040	AP023338.1
☑	Escherichia coli strain TD-33 plasmid pNDM-TD33, complete sequence	Escherichia coli	13308	22350	99%	0.0	99.81%	141890	MN915013.1
☑	Escherichia coli strain GD-33 plasmid pNDM33-1, complete sequence	Escherichia coli	13308	28018	99%	0.0	99.81%	266777	MN915011.1
☑	Escherichia coli strain TJ-33 plasmid pNDM-TJ33, complete sequence	Escherichia coli	13308	31030	99%	0.0	99.81%	366267	MN915010.1
☑	Klebsiella pneumoniae strain AR_0125 plasmid tig00000005_pilon, complete sequence	Klebsiella pneu…	13308	14819	99%	0.0	99.81%	47277	CP021858.1
☑	Klebsiella pneumoniae strain AR_0113 plasmid unitig_4, complete sequence	Klebsiella pneu…	13308	14825	99%	0.0	99.81%	45740	CP021755.1
☑	Klebsiella pneumoniae strain AR_0112 plasmid tig00000003, complete sequence	Klebsiella pneu…	13308	14825	99%	0.0	99.81%	45740	CP021547.1
☑	Klebsiella pneumoniae strain AR_0115 plasmid tig00000003, complete sequence	Klebsiella pneu…	13308	13308	99%	0.0	99.81%	39762	CP020073.1
☑	Klebsiella pneumoniae strain NY9, complete genome	Klebsiella pneu…	13308	14817	99%	0.0	99.81%	5348589	CP015385.1
☑	Klebsiella pneumoniae strain CAV1596 plasmid pCAV1596-41, complete sequence	Klebsiella pneu…	13308	13308	99%	0.0	99.81%	40939	CP011644.1
☑	Salmonella sp. SAL-045 plasmid unnamed1, complete sequence	Salmonella sp.…	13308	28397	99%	0.0	99.81%	318041	CP071694.1
☑	[plasmid=p33Kpn22-5]	Klebsiella pneu…	13308	14825	99%	0.0	99.81%	47426	CP069051.1
☑	Klebsiella quasipneumoniae plasmid pHN111WT-1, complete sequence	Klebsiella quasi…	13308	16948	99%	0.0	99.81%	220731	MT647839.1
☑	Salmonella enterica subsp. enterica serovar Typhimurium strain S304 plasmid pS304_1, complete sequence	Salmonella ent…	13308	25375	99%	0.0	99.81%	254873	CP061127.1
☑	Klebsiella pneumoniae strain 2019036D plasmid p2019036D-50kb, complete sequence	Klebsiella pneu…	13302	17837	99%	0.0	99.79%	50845	CP047338.1
☑	Klebsiella pneumoniae strain KP18-29 plasmid p18-29-MDR, complete sequence	Klebsiella pneu…	13302	18695	99%	0.0	99.79%	278636	MK262712.1
☑	Klebsiella pneumoniae strain ESBL_DR09 plasmid pESBL_DR09b, complete sequence	Klebsiella pneu…	13302	17826	99%	0.0	99.79%	65548	CP067257.1

Figure S1. BLAST analysis of 7259 bp sequence

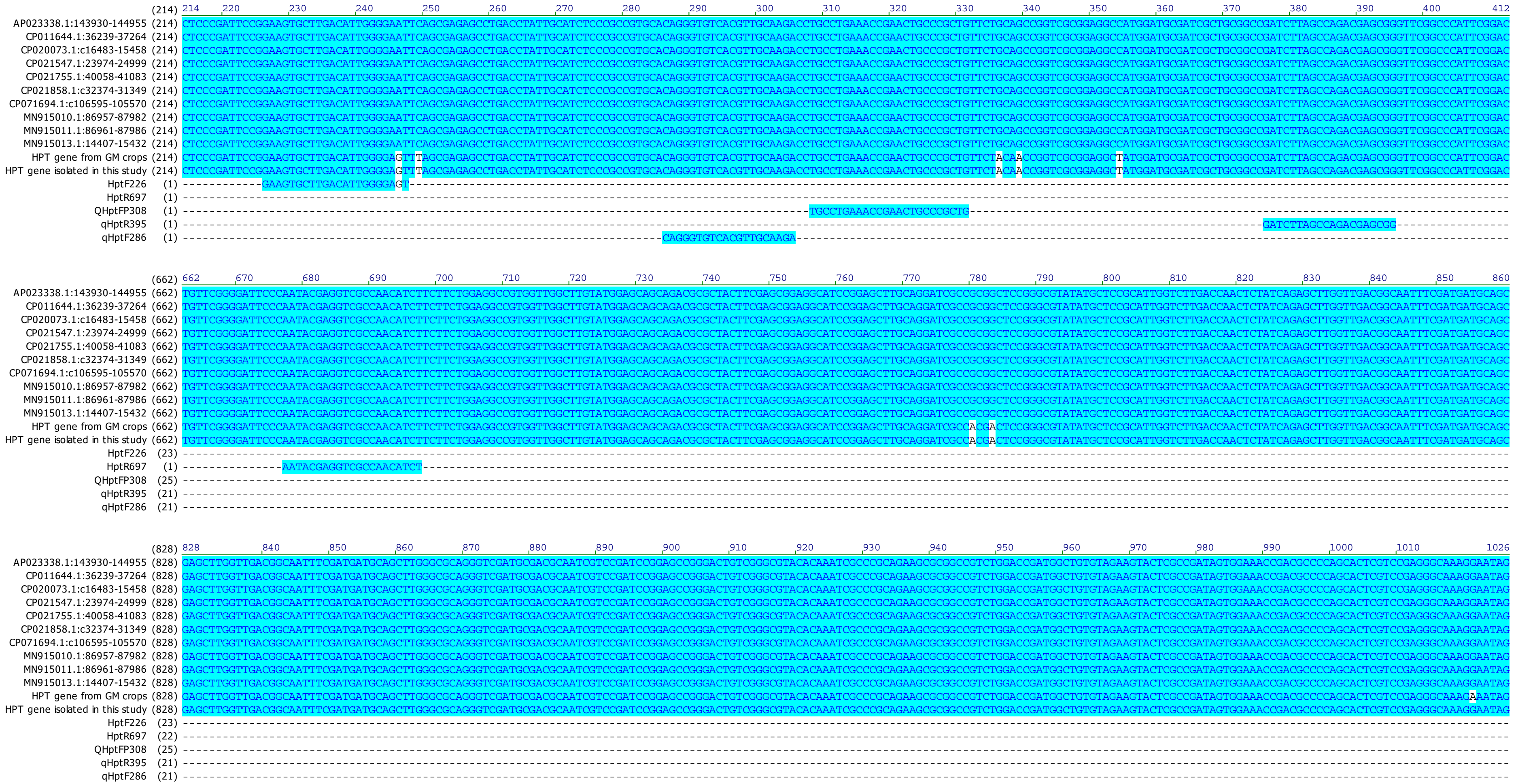

Figure S2. Alignment of *HPT* genes from the plasmid DNA of microorganisms, GM crops and isolated sequence in this study.

```
                 (1) 1        10        20        30        40        50        60        70        80        90        100       110       120       130       140       150       160       171
  AP023338.1     (1) MKKPELTATSVEKFLIEKFDSVSDLMQLSEGEESRAFSFDVGGRGYVLRVNSCADGFYKDRYVYRHFASAALPIPEVLDIGEFSESLTYCISRRAQGVTLQDLPETELPAVLQPVAEAMDAIAAADLSQTSGFGPFGPQGIGQYTTWRDFICAIADPHVYHWQTVMDDTVS
  CP011644.1     (1) MKKPELTATSVEKFLIEKFDSVSDLMQLSEGEESRAFSFDVGGRGYVLRVNSCADGFYKDRYVYRHFASAALPIPEVLDIGEFSESLTYCISRRAQGVTLQDLPETELPAVLQPVAEAMDAIAAADLSQTSGFGPFGPQGIGQYTTWRDFICAIADPHVYHWQTVMDDTVS
  CP021547.1     (1) MKKPELTATSVEKFLIEKFDSVSDLMQLSEGEESRAFSFDVGGRGYVLRVNSCADGFYKDRYVYRHFASAALPIPEVLDIGEFSESLTYCISRRAQGVTLQDLPETELPAVLQPVAEAMDAIAAADLSQTSGFGPFGPQGIGQYTTWRDFICAIADPHVYHWQTVMDDTVS
CP021547.1#2     (1) MKKPELTATSVEKFLIEKFDSVSDLMQLSEGEESRAFSFDVGGRGYVLRVNSCADGFYKDRYVYRHFASAALPIPEVLDIGEFSESLTYCISRRAQGVTLQDLPETELPAVLQPVAEAMDAIAAADLSQTSGFGPFGPQGIGQYTTWRDFICAIADPHVYHWQTVMDDTVS
  CP021755.1     (1) MKKPELTATSVEKFLIEKFDSVSDLMQLSEGEESRAFSFDVGGRGYVLRVNSCADGFYKDRYVYRHFASAALPIPEVLDIGEFSESLTYCISRRAQGVTLQDLPETELPAVLQPVAEAMDAIAAADLSQTSGFGPFGPQGIGQYTTWRDFICAIADPHVYHWQTVMDDTVS
  CP021858.1     (1) MKKPELTATSVEKFLIEKFDSVSDLMQLSEGEESRAFSFDVGGRGYVLRVNSCADGFYKDRYVYRHFASAALPIPEVLDIGEFSESLTYCISRRAQGVTLQDLPETELPAVLQPVAEAMDAIAAADLSQTSGFGPFGPQGIGQYTTWRDFICAIADPHVYHWQTVMDDTVS
  CP071694.1     (1) MKKPELTATSVEKFLIEKFDSVSDLMQLSEGEESRAFSFDVGGRGYVLRVNSCADGFYKDRYVYRHFASAALPIPEVLDIGEFSESLTYCISRRAQGVTLQDLPETELPAVLQPVAEAMDAIAAADLSQTSGFGPFGPQGIGQYTTWRDFICAIADPHVYHWQTVMDDTVS
  MN915010.1     (1) MKKPELTATSVEKFLIEKFDSVSDLMQLSEGEESRAFSFDVGGRGYVLRVNSCADGFYKDRYVYRHFASAALPIPEVLDIGEFSESLTYCISRRAQGVTLQDLPETELPAVLQPVAEAMDAIAAADLSQTSGFGPFGPQGIGQYTTWRDFICAIADPHVYHWQTVMDDTVS
  MN915011.1     (1) MKKPELTATSVEKFLIEKFDSVSDLMQLSEGEESRAFSFDVGGRGYVLRVNSCADGFYKDRYVYRHFASAALPIPEVLDIGEFSESLTYCISRRAQGVTLQDLPETELPAVLQPVAEAMDAIAAADLSQTSGFGPFGPQGIGQYTTWRDFICAIADPHVYHWQTVMDDTVS
  MN915013.1     (1) MKKPELTATSVEKFLIEKFDSVSDLMQLSEGEESRAFSFDVGGRGYVLRVNSCADGFYKDRYVYRHFASAALPIPEVLDIGEFSESLTYCISRRAQGVTLQDLPETELPAVLQPVAEAMDAIAAADLSQTSGFGPFGPQGIGQYTTWRDFICAIADPHVYHWQTVMDDTVS
        HPT1     (1) MKKPELTATSVEKFLIEKFDSVSDLMQLSEGEESRAFSFDVGGRGYVLRVNSCADGFYKDRYVYRHFASAALPIPEVLDIGEFSESLTYCISRRAQGVTLQDLPETELPAVLQPVAEAMDAIAAADLSQTSGFGPFGPQGIGQYTTWRDFICAIADPHVYHWQTVMDDTVS
        HPT2     (1) MKKPELTATSVEKFLIEKFDSVSDLMQLSEGEESRAFSFDVGGRGYVLRVNSCADGFYKDRYVYRHFASAALPIPEVLDIGEFSESLTYCISRRAQGVTLQDLPETELPAVLQPVAEAMDAIAAADLSQTSGFGPFGPQGIGQYTTWRDFICAIADPHVYHWQTVMDDTVS

               (172) 172      180       190       200       210       220       230       240       250       260       270       280       290       300       310       320       330       341
  AP023338.1   (172) ASVAQALDELMLWAEDCPEVRHLVHADFGSNNVLTDNGRITAVIDWSEAMFGDSQYEVANIFFWRPWLACMEQQTRYFERRHPELAGSPRLRAYMLRIGLDQLYQSLVDGNFDDAAWAQGRCDAIVRSGAGTVGRTQIARRSAAVWTDGCVEVLADSGNRRPSTRPRAKE
  CP011644.1   (172) ASVAQALDELMLWAEDCPEVRHLVHADFGSNNVLTDNGRITAVIDWSEAMFGDSQYEVANIFFWRPWLACMEQQTRYFERRHPELAGSPRLRAYMLRIGLDQLYQSLVDGNFDDAAWAQGRCDAIVRSGAGTVGRTQIARRSAAVWTDGCVEVLADSGNRRPSTRPRAKE
  CP021547.1   (172) ASVAQALDELMLWAEDCPEVRHLVHADFGSNNVLTDNGRITAVIDWSEAMFGDSQYEVANIFFWRPWLACMEQQTRYFERRHPELAGSPRLRAYMLRIGLDQLYQSLVDGNFDDAAWAQGRCDAIVRSGAGTVGRTQIARRSAAVWTDGCVEVLADSGNRRPSTRPRAKE
CP021547.1#2   (172) ASVAQALDELMLWAEDCPEVRHLVHADFGSNNVLTDNGRITAVIDWSEAMFGDSQYEVANIFFWRPWLACMEQQTRYFERRHPELAGSPRLRAYMLRIGLDQLYQSLVDGNFDDAAWAQGRCDAIVRSGAGTVGRTQIARRSAAVWTDGCVEVLADSGNRRPSTRPRAKE
  CP021755.1   (172) ASVAQALDELMLWAEDCPEVRHLVHADFGSNNVLTDNGRITAVIDWSEAMFGDSQYEVANIFFWRPWLACMEQQTRYFERRHPELAGSPRLRAYMLRIGLDQLYQSLVDGNFDDAAWAQGRCDAIVRSGAGTVGRTQIARRSAAVWTDGCVEVLADSGNRRPSTRPRAKE
  CP021858.1   (172) ASVAQALDELMLWAEDCPEVRHLVHADFGSNNVLTDNGRITAVIDWSEAMFGDSQYEVANIFFWRPWLACMEQQTRYFERRHPELAGSPRLRAYMLRIGLDQLYQSLVDGNFDDAAWAQGRCDAIVRSGAGTVGRTQIARRSAAVWTDGCVEVLADSGNRRPSTRPRAKE
  CP071694.1   (172) ASVAQALDELMLWAEDCPEVRHLVHADFGSNNVLTDNGRITAVIDWSEAMFGDSQYEVANIFFWRPWLACMEQQTRYFERRHPELAGSPRLRAYMLRIGLDQLYQSLVDGNFDDAAWAQGRCDAIVRSGAGTVGRTQIARRSAAVWTDGCVEVLADSGNRRPSTRPRAKE
  MN915010.1   (172) ASVAQALDELMLWAEDCPEVRHLVHADFGSNNVLTDNGRITAVIDWSEAMFGDSQYEVANIFFWRPWLACMEQQTRYFERRHPELAGSPRLRAYMLRIGLDQLYQSLVDGNFDDAAWAQGRCDAIVRSGAGTVGRTQIARRSAAVWTDGCVEVLADSGNRRPSTRPRAKE
  MN915011.1   (172) ASVAQALDELMLWAEDCPEVRHLVHADFGSNNVLTDNGRITAVIDWSEAMFGDSQYEVANIFFWRPWLACMEQQTRYFERRHPELAGSPRLRAYMLRIGLDQLYQSLVDGNFDDAAWAQGRCDAIVRSGAGTVGRTQIARRSAAVWTDGCVEVLADSGNRRPSTRPRAKE
  MN915013.1   (172) ASVAQALDELMLWAEDCPEVRHLVHADFGSNNVLTDNGRITAVIDWSEAMFGDSQYEVANIFFWRPWLACMEQQTRYFERRHPELAGSPRLRAYMLRIGLDQLYQSLVDGNFDDAAWAQGRCDAIVRSGAGTVGRTQIARRSAAVWTDGCVEVLADSGNRRPSTRPRAKE
        HPT1   (172) ASVAQALDELMLWAEDCPEVRHLVHADFGSNNVLTDNGRITAVIDWSEAMFGDSQYEVANIFFWRPWLACMEQQTRYFERRHPELAGSPRLRAYMLRIGLDQLYQSLVDGNFDDAAWAQGRCDAIVRSGAGTVGRTQIARRSAAVWTDGCVEVLADSGNRRPSTRPRAKE
        HPT2   (172) ASVAQALDELMLWAEDCPEVRHLVHADFGSNNVLTDNGRITAVIDWSEAMFGDSQYEVANIFFWRPWLACMEQQTRYFERRHPELAGSPRLRAYMLRIGLDQLYQSLVDGNFDDAAWAQGRCDAIVRSGAGTVGRTQIARRSAAVWTDGCVEVLADSGNRRPSTRPRAKK
```

Figure S3. Amino acid alignment of HPT from the plasmid DNA of microorganisms, GM crops and isolated sequence in this study. HPT1: Amino acid sequence of HPT isolated in this study. HPT2: Amino acid sequence of HPT from GM crops.

Description	Scientific Name	Max Score	Total Score	Query Cover	E value	Per. Ident	Acc. Len	Accession
Escherichia coli strain AH25 plasmid pAH25-2, complete sequence	Escherichia coli	483	483	100%	4e-132	100.00%	116030	CP055258.1
Escherichia coli strain AH01 plasmid pAH01-4, complete sequence	Escherichia coli	483	483	100%	4e-132	100.00%	145790	CP055255.1
Proteus mirabilis strain MPE5139 chromosome, complete genome	Proteus mirabilis	483	483	100%	4e-132	100.00%	4104163	CP053684.1
Proteus mirabilis strain MPE0027 chromosome, complete genome	Proteus mirabilis	483	958	100%	4e-132	100.00%	4120711	CP053683.1
Proteus mirabilis strain MPE0734 chromosome, complete genome	Proteus mirabilis	483	483	100%	4e-132	100.00%	4055574	CP053615.1
Proteus mirabilis strain MPE0767 chromosome, complete genome	Proteus mirabilis	483	483	100%	4e-132	100.00%	4055642	CP053616.1
Proteus mirabilis strain YPM35 chromosome, complete genome	Proteus mirabilis	483	483	100%	4e-132	100.00%	4161306	CP053898.1
Proteus mirabilis strain JPM24 chromosome, complete genome	Proteus mirabilis	483	483	100%	4e-132	100.00%	3983870	CP053894.1
Salmonella enterica subsp. enterica serovar Infantis strain CVM N17S1509 plasmid pN17S1509, complete se...	Salmonella ent...	483	483	100%	4e-132	100.00%	306853	CP052818.1
Klebsiella pneumoniae VNCKp115 plasmid pVNCKp115 DNA, complete sequence	Klebsiella pneu...	483	483	100%	4e-132	100.00%	298683	LC549807.1
Proteus terrae subsp. cibarius strain HNCF43W chromosome, complete genome	Proteus terrae...	483	483	100%	4e-132	100.00%	3965977	CP053044.1
Enterobacter sp. isolate TAir plasmid pTAir-5	Enterobacter sp.	483	483	100%	4e-132	100.00%	49172	CP063952.1
Escherichia coli strain EC11 plasmid plas1-MCR, complete sequence	Escherichia coli	483	483	100%	4e-132	100.00%	223103	CP073361.1
Proteus mirabilis strain S62-3-2-2 chromosome, complete genome	Proteus mirabilis	483	483	100%	4e-132	100.00%	3952956	CP073247.1
Proteus mirabilis strain 1035 chromosome, complete genome	Proteus mirabilis	483	483	100%	4e-132	100.00%	4156152	CP072779.1
Escherichia coli strain ECONC427 plasmid p1_ECONC427, complete sequence	Escherichia coli	483	483	100%	4e-132	100.00%	233336	CP071135.1
Bacterium RGF134-1 strain RGF134-1 chromosome, complete genome	bacterium RGF...	483	483	100%	4e-132	100.00%	4095724	CP066833.1
Klebsiella pneumoniae strain ESBL_DR09 plasmid pESBL_DR09b, complete sequence	Klebsiella pneu...	483	483	100%	4e-132	100.00%	65548	CP067257.1
Escherichia coli strain EC014 plasmid pEC014-2, complete sequence	Escherichia coli	483	483	100%	4e-132	100.00%	154516	MW317021.1
Salmonella sp. SAL-045 plasmid unnamed1, complete sequence	Salmonella sp....	483	483	100%	4e-132	100.00%	318041	CP071694.1
Klebsiella pneumoniae strain IR5755 chromosome, complete genome	Klebsiella pneu...	483	483	100%	4e-132	100.00%	5447830	CP061970.1
Escherichia coli strain LZYWN_4 plasmid pS4-aph, complete sequence	Escherichia coli	483	483	100%	4e-132	100.00%	113995	CP046417.1
Salmonella enterica strain GSJ/2016-Sal.-018 plasmid pSal018, complete sequence	Salmonella ent...	483	483	100%	4e-132	100.00%	217773	CP069167.1

Figure S4. BLAST analysis of 261 bp amplified sequence
